# Regulation of Nicotine Tolerance by Quorum Sensing and High Efficiency of Quorum Quenching Under Nicotine Stress in *Pseudomonas aeruginosa* PAO1

**DOI:** 10.3389/fcimb.2018.00088

**Published:** 2018-03-20

**Authors:** Huiming Tang, Yunyun Zhang, Yifan Ma, Mengmeng Tang, Dongsheng Shen, Meizhen Wang

**Affiliations:** ^1^School of Environmental Science and Engineering, Zhejiang Gongshang University, Hangzhou, China; ^2^Zhejiang Provincial Key Laboratory of Solid Waste Treatment and Recycling, Hangzhou, China

**Keywords:** nicotine tolerance, quorum sensing, antioxidant-producing ability, biofilm formation, quorum quenching, virulence

## Abstract

Quorum sensing (QS) regulates the behavior of bacterial populations and promotes their adaptation and survival under stress. As QS is responsible for the virulence of vast majority of bacteria, quorum quenching (QQ), the interruption of QS, has become an attractive therapeutic strategy. However, the role of QS in stress tolerance and the efficiency of QQ under stress in bacteria are seldom explored. In this study, we demonstrated that QS-regulated catalase (CAT) expression and biofilm formation help *Pseudomonas aeruginosa* PAO1 resist nicotine stress. CAT activity and biofilm formation in wild type (WT) and Δ*rhlR* strains are significantly higher than those in the Δ*lasR* strain. Supplementation of Δ*lasI* strain with 3OC12-HSL showed similar CAT activity and biofilm formation as those of the WT strain. LasIR circuit rather than RhlIR circuit is vital to nicotine tolerance. Acylase I significantly decreased the production of virulence factors, namely elastase, pyocyanin, and pyoverdine under nicotine stress compared to the levels observed in the absence of nicotine stress. Thus, QQ is more efficient under stress. To our knowledge, this is the first study to report that QS contributes to nicotine tolerance in *P. aeruginosa*. This work facilitates a better application of QQ for the treatment of bacterial infections, especially under stress.

## Introduction

Cell density-dependent cell-to-cell communication, termed as quorum sensing (QS), regulates the behavior of bacterial populations (Waters and Bassler, 2005). Bacteria secrete and share QS signaling molecules that bind to cognate receptors, and upon reaching critical concentration induce cell density-dependent adaptive responses within the population (Albuquerque et al., 2014). QS is responsible for a number of collective behavioral properties, including virulence factor secretion, biofilm formation, and horizontal gene transfer (Antonova and Hammer, 2011; Joo and Otto, 2012; Yang et al., 2017). Compared to individuality, sociality, regulated by QS, significantly increases the bacterial fitness in various environment (Darch et al., 2012). Despite increasing recognition on bacterial QS, the roles that they play in the response of environmental stress are far from fully understood (García-contreras et al., 2015).

Quorum sensing (QS) regulates the secretion of virulence factors from a broad spectrum of bacterial pathogens, including *Pseudomonas aeruginosa* (De Kievit and Iglewski, 2000). QS also participates in the development of biofilms, which are responsible for resistance to antibiotics, in many infections (Hazan et al., 2016). Due to the role of QS in pathogenicity and antibiotic resistance, the different factors involved in these pathways are considered to be attractive targets for novel antimicrobial agents (Starkey et al., 2014; Wang et al., 2016; Whiteley et al., 2017). Interruption of QS, which is known as quorum quenching (QQ), has been explored to control bacterial pathogenicity (Chan et al., 2015). As QS is an active process in response to environmental changes, QQ will have to be applicable under various conditions. Therefore, analysis of the QS response under different environmental conditions is vital for developing an efficient strategy involving QQ to control pathogenicity of bacteria.

*Pseudomonas aeruginosa*, one of the most common pathogenic bacteria in the world, not only infects humans, but also plants (Valentini et al., 2017). Its pathogenicity is mainly regulated by QS (Girard and Bloemberg, 2008; Whiteley et al., 2017). *P. aeruginosa* has two acyl-homoserine lactones (AHLs) QS circuits, LasIR and RhlIR (Stover et al., 2000). In LasIR circuit, LasI catalyzes the synthesis of *N*-3-oxo-dodecanoyl homoserine lactone (3OC12-HSL), which binds to its cognate receptor LasR and subsequently induces the expression of elastase-encoding genes involved in the development of pathogenicity of the bacteria (Pearson et al., 1994). For RhlIR circuit, RhlI catalyzes the synthesis of butyryl-HSL (C4-HSL), which binds to RhlR and subsequently activates a series of virulence factors including pyocyanin (Mukherjee et al., 2017). The well-elucidated mechanism of QS in *P. aeruginosa* allows us to study the feasibility of applying QQ to reduce the pathogenicity of the bacteria.

Though *P. aeruginosa* causes infection in both, humans and plants, they are exposed to various conditions. *P. aeruginosa* is known to inhabit hypoxic mucus plugs in the lungs of cystic fibrosis (CF) patient. Nearly 30% of smokers were involved in the population of CF patient (Ortega-García et al., 2012). In addition, the growth of *P. aeruginosa* in stems and rots leads to systemic infection and ultimately to the development of severe soft-rot symptoms in tobacco (Pfeilmeier et al., 2016). Nicotine is one of the main alkaloid in tobacco. Recent evidence has demonstrated that *P. aeruginosa* could grow under nicotine stress in tobacco plants or human being, but few studies regarding the role of QS in nicotine tolerance in *P. aeruginosa* have been performed (Hutcherson et al., 2015), limiting the development and application of strategies involving QQ to control its pathogenicity under nicotine-stress conditions.

Thus, we employed *P. aeruginosa* PAO1 as the model bacteria and nicotine as the typical stress. First, the growth and antioxidant-producing and biofilm-formation ability of wild-type (WT) strains and their signal-blind mutants were compared to investigate the role of QS in nicotine tolerance. Second, competition assay under nicotine stress and complementation experiment using a signal-deficient mutant were performed to analyze the possible mechanism. Finally, the efficiency of a QS inhibitor was analyzed under the presence and absence of nicotine stress to evaluate the application of QQ under these conditions. To our knowledge, this is the first study to report that QS plays an important role in nicotine tolerance, and demonstrates that LasIR circuit, rather than the RhlIR circuit, is responsible for nicotine tolerance in *P. aeruginosa* PAO1. This information will help to improve our understanding of the role of bacterial QS under stress, and to develop and apply QQ-based strategies for combating bacterial infection in the future.

## Materials and methods

### Bacterial strains, media, and culture

The bacterial strains used in this study were *P. aeruginosa* PAO1 WT strain and its QS mutants Δ*lasR*, Δ*rhlR*, and Δ*lasI* (Wang et al., 2015).

Luria-Bertani (LB) medium with or without nicotine was used in this study. LB medium was composed of tryptone (10 g), yeast extract (5 g), NaCl (5 g) in 1 L distilled water. Filtered-sterile nicotine (0–2.0 g/L) was replenished according to requirement.

Inocula were obtained from overnight LB cultures. The initial optical density (OD) was 0.001 (600 nm), except where noted. The culture was incubated in a shaker, at 37°C with 250 rpm.

### The detection of reactive oxygen species (ROS)

Wildtype strain, PAO1, was inoculated into LB with initial OD_600_ of 0.01. After the growth of the cells entered the logarithmic phase (OD_600_ = 1), 0, 1.6, and 2.0 g/L nicotine was added into the culture. To measure ROS, 2′,7′-dichlorofluorescin diacetate (DCFH-DA) was added at a final concentration of 10 mM. Within 1 h of incubation, DCFH-DA was hydrolyzed into dichlorofluorescin (DCFH) in the cells. Then DCFH was oxidized by ROS into dichlorofluorescein (DCF). DCF was measured using SpectraMax® i3 plate reader at 488 nm of excitation and 525 nm of emission (Molecular Devices, Sunnyvale, CA, USA) (Yu et al., 2014). H_2_O_2_ treatment was used as a positive control. We calculated the relative ROS level by dividing the value of the DCF level obtained for experimental samples by that for LB medium.

### The measurement of the activity of catalase (CAT) and superoxide dismutase (SOD)

After exposure to 0, 1.6, and 2.0 g/L of nicotine, cells in logarithmic phase were harvested to detect the activity of CAT and SOD, respectively. Cells were washed thrice with 0.9% NaCl and ultrasonically lysed. Subsequently, crude enzymes were obtained by centrifugation at 4°C and 12,000 rpm for 10 min. The activity of CAT and SOD was detected using the ammonium molybdate method (A007) and hydroxylamine method (A001-1-1), respectively. The total protein content was determined using a modified Bradford assay (Kit A045). All assays were performed according to manufacturer's instructions. These kits were purchased from the Nanjing Jiancheng Bioengineering Institute (Jiangsu, China).

One unit of CAT activity was defined as the amount of lysate that catalyzes the decomposition of 1 μM of H_2_O_2_ per minute at 37°C. One unit of SOD activity was defined as the amount of lysate that inhibits the rate of xanthine/xanthine oxidase-dependent cytochrome-c reduction at 25°C by 50%. The activities of both enzymes were expressed as units per mg of cellular protein.

### Biofilm formation analysis

After exposure to 0, 1.6, and 2.0 g/L of nicotine, the biofilm formation in 10-mL tubes was evaluated. Biofilm biomass was analyzed by crystal violet (CV) staining method described by Wang et al. (2012). After 24 h of incubation, the tubes were carefully washed twice with phosphate-buffered saline (PBS) to remove planktonic cells. After air drying for 5 min, biofilms were stained with 1 mL of 0.1% CV for 10 min, then the tubes were rinsed thoroughly thrice with distilled water to remove the unabsorbed CV. Finally, adhered CV was solubilized with 3 mL of alcohol acetone (4:1, v/v) and measured at 570 nm using a SpectraMax® i3 plate reader (Molecular Devices, Sunnyvale, CA, USA).

The polysaccharides, protein and DNA component of biofilm was analyzed according to Wang et al. (2012). In brief, the biofilm was washed thrice and resuspended in PBS. Subsequently, the suspension was heated to 80°C for 45 min, and the mixture was centrifuged at 13,000 rpm for 20 min to remove solid residues. The extracellular polysaccharides (EPS) and extracellular protein as the two main components of biofilm were determined using the phenol/sulfuric acid method (Dubois et al., 1956) and Coomassie brilliant blue assay (Bradford, 1976), respectively. The content of extracellular DNA as the other component of biofilm was quantified using a Nano-drop 2000 spectrophotometer after purification with a phenol/chloroform/isoamyl reagent.

The morphology of biofilm was observed by confocal laser scanning microscopy (CLSM, Leica, Germany). For ease of observation, crude glass slides were placed in flasks containing 0, 1.6, and 2.0 g/L of nicotine, and biofilms formed on these slides. The cell viability in biofilm was determined using a double live/dead staining kit containing nucleic acid stains SYTO 9 and propidium iodide (PI). After biofilm formation, the glass slides were gently rinsed by immersing them in PBS, removing all unadhered cells, and subsequently, stained for 15 min. Viable bacteria with intact cell membrane were stained with green, whereas dead bacteria with damaged membrane were stained with red. Stained samples were visualized with the following excitation/emission detectors and filter sets: for SYTO 9, 480/500 and for PI, 490/635 (Shi et al., 2016).

### Coculture assay

WT, Δ*lasR*, and Δ*rhlR* strains were grown to mid-logarithmic phase, respectively. WT *vs*. Δ*lasR*, and Δ*rhlR vs*. Δ*lasR* with the ratio of 1:1 (cell number) were separately cocultured in LB media with 0, 0.4, 0.8, 1.2, 1.6, and 2.0 g/L nicotine under 37°C for 24 h. The initial OD_600_ was 0.05. Then, skim milk agars were used to differentiate the Δ*lasR* strains from WT or Δ*rhlR* strains, where a clear zone appeared around WT and Δ*rhlR* colonies but not around Δ*lasR* colonies (Wang et al., 2015). Skim milk agar was prepared as follows (/L): 1.25 g NaCl, 1.25 g yeast extract, 2.5 g tryptone, 80 g skim milk powder, and 15 g agar. For each value reported, at least 300 colonies were screened.

### QQ assay

Acylase I (Kit A8376-1G, Sigma, Germany) was used for QQ (Yeon et al., 2008) Overnight culture of the WT strain was inoculated into LB with 0, 1.6, and 2.0 g/L of nicotine. After 12 h of incubation, 0.25 mg/L acylase I was replenished to interrupt both, 3OC12-HSL and C4-HSL-mediated QS circuits. After another 12 h of incubation, the production of QS-regulated products including elastase, pyocyanin, and pyoverdine was compared among different culture conditions.

Elastase was detected by Pierce Fluorescent Protease Assay kit (Thermo). In brief, the culture was centrifuged at 12,000 rpm for 15 min. Subsequently, 100 μL of the supernatant was mixed with 100 μL of succinylated-casein solution (1:500 mixture of 2 g/L lyophilized succinylated casein and trinitrobenzene sulfonic acid, pH = 8.5) and incubated for 45 min in the dark at room temperature. The fluorescence was detected at 450 nm using a plate reader (SpectraMax® i3, Molecular Devices, Sunnyvale, CA, USA).

Pyocyanin was measured by chloroform and hydrochloric acid extraction (Pearson et al., 1994). A total of 1.5 mL of chloroform was used to extract 2.5 mL of the supernatant. The pyocyanin was re-extracted from the chloroform using 1 mL of 0.2 M hydrochloric acid. Finally, the absorbance of the supernatant was measured at 520 nm. The concentration of pyocyanin was equal to the absorbance multiplied by 12.8 mg/L.

Pyoverdine was detected using the method described by Wurst et al. (2014). In brief, the cultures were centrifuged at 12,000 rpm for 15 min. The absorbance of the supernatant was measured at 405 nm.

The level of elastase, pyocyanin, and pyoverdine were expressed as units per OD_600_ unit in order to avoid the interference of cell density. All experiments were in triplicate.

### Statistical analysis

GraphPad Prism 6.0 software was used for statistical analyses. Two-way ANOVA and *t*-test were performed. Differences with a value of *p* < 0.05 were considered to be statistically significant.

## Results

### QS plays an important role in nicotine tolerance

QS is involved in the regulation of the behavior of a bacterial population, whereby the cells secrete diffusible substances that generate phenotypic responses in the living group. Compared to individuality, sociality confers a 100–1,000-fold increase in resistance to stress (Hazan et al., 2016). Thus, our hypothesis is that QS possibly plays an important role in nicotine tolerance. To confirm this hypothesis, a simple experiment comparing the growth of the WT strain with complete QS circuits and the signal-blind mutants under nicotine stress, was performed. Signal-blind mutants cannot respond to their cognate signals, and therefore, the expression of their corresponding regulons is inhibited.

**Figure 1:**
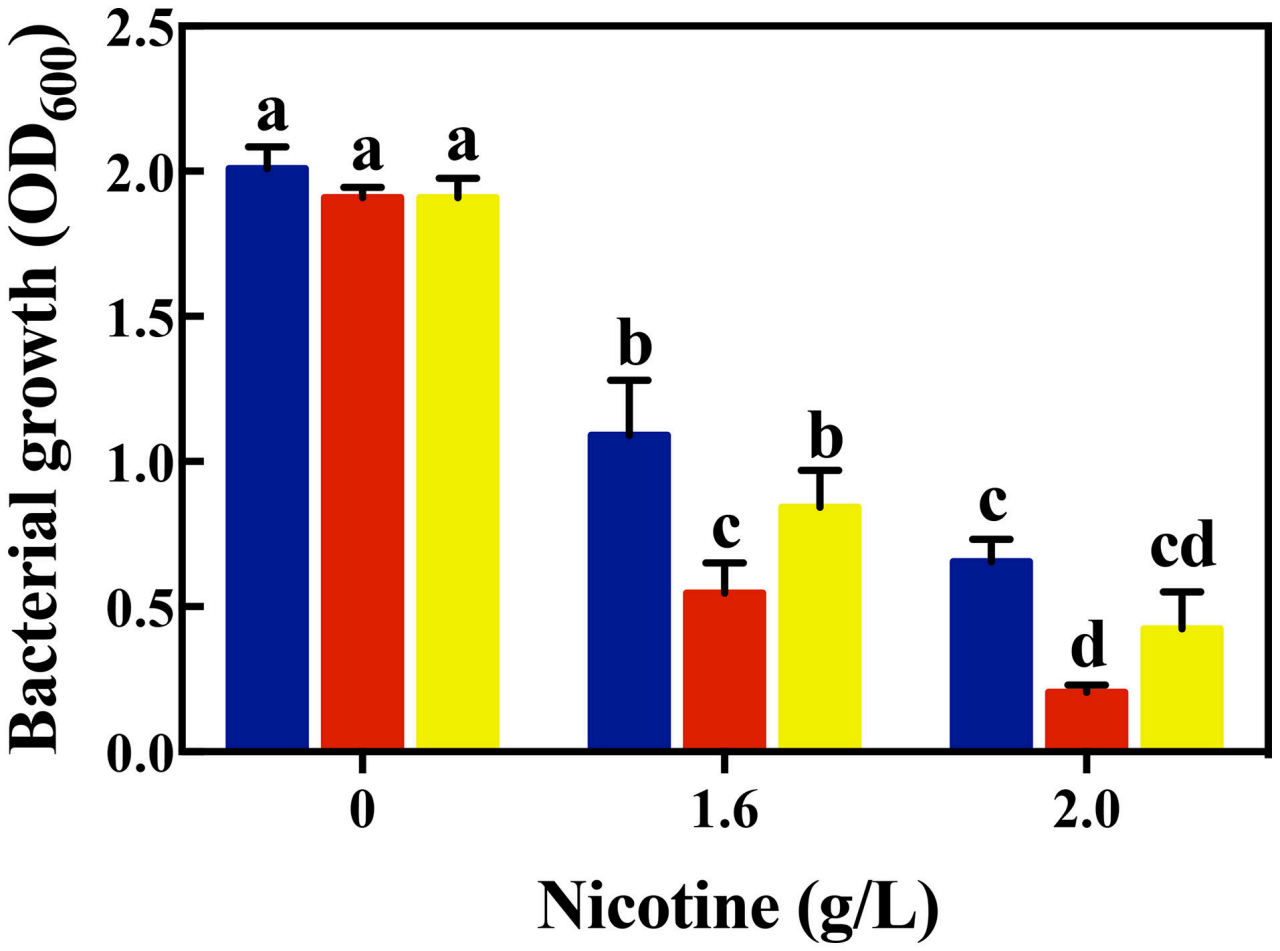
Comparison of bacterial growth among different strains (WT, blue bars; Δ*lasR*, red bars; Δ*rhlR*, yellows bars) under exposure to different concentrations of nicotine. Different letters indicate significant difference at *p* < 0.05 and the same letter indicates no significant difference.

As shown in Figure 1, there was no difference of bacterial growth between the WT and signal-blind mutant Δ*lasR and* Δ*rhlR* strains in the absence of nicotine. Under a 1.6 g/L-nicotine treatment, the growth of the WT, Δ*lasR, and* Δ*rhlR* strains was inhibited. However, the growth of the Δ*lasR* strain was significantly lower than that of the WT and Δ*rhlR* strains. Similar to the result of the 1.6 g/L-nicotine treatment, the growth of all three strains was inhibited under a 2.0 g/L-nicotine treatment. The lowest growth was observed in Δ*lasR* culture. Though other mechanisms possibly exist, the results indicated that QS played an important role in nicotine tolerance by *P. aeruginosa* PAO1.

### Antioxidant ability regulated by QS benefit for nicotine tolerance

Nicotine is a carcinogenic, teratogenic, and mutagenic substance, which can induce the production of a large number of free radicals, resulting in oxidative damage to cells (Haussmann and Fariss, 2016). The comparison of bacterial growth indicated that QS played an important role in nicotine tolerance. According to García-contreras et al. (2015), QS is able to exert a robust anti-oxidative response. Thus, one possibility could be that the role of QS in anti-oxidative response was beneficial for nicotine tolerance.

**Figure 2:**
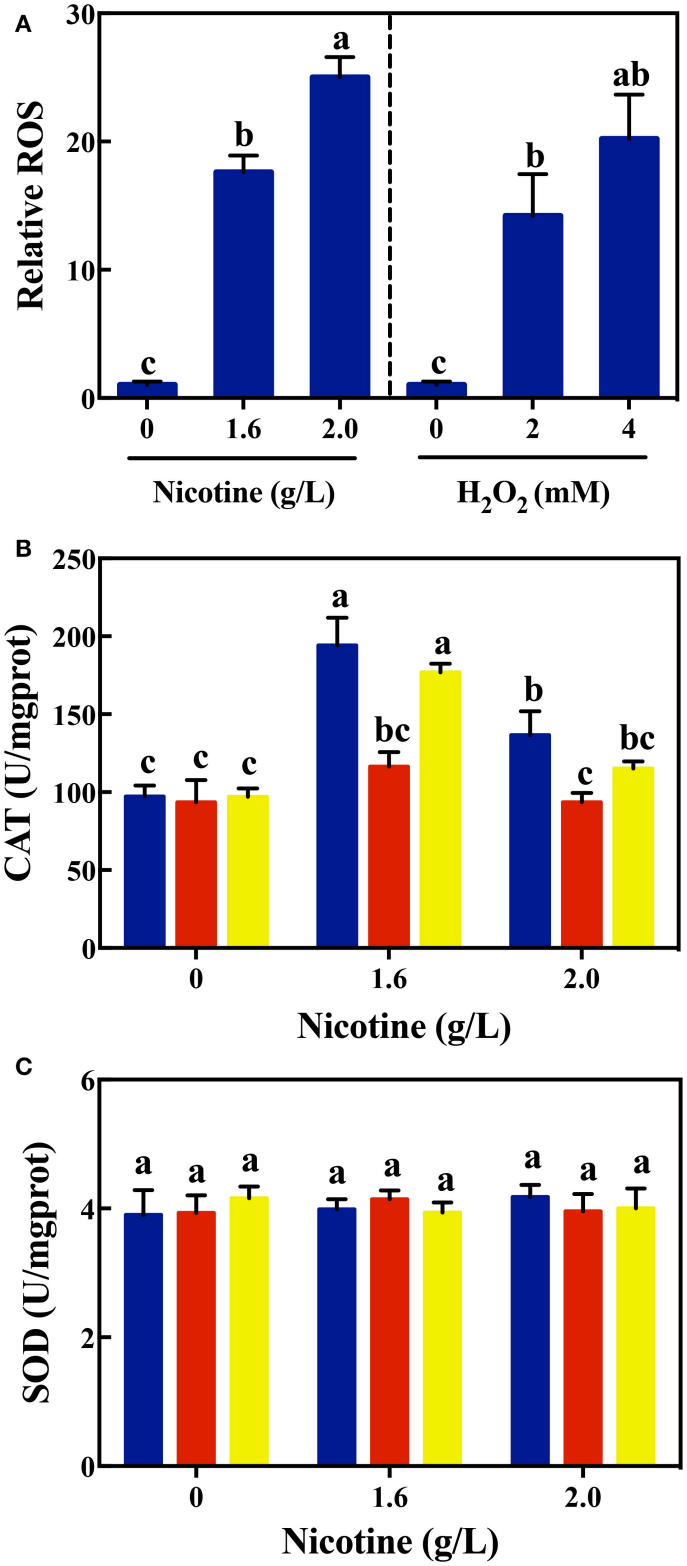
Relative ROS levels **(A)** in WT on exposure to different concentrations of nicotine (left) and H_2_O_2_ (right); CAT activity **(B)** and SOD activity **(C)** among different strains (WT, blue bars; Δ*lasR*, red bars; Δ*rhlR*, yellows bars) under exposure to different concentrations of nicotine. Different letters indicate significant difference at *p* < 0.05 and the same letter indicates no significant difference.

In order to validate this assumption, we first evaluated the ROS generation under nicotine exposure. As shown in Figure 2A, the level of intracellular ROS in WT cells increased significantly with the increase in nicotine. Nicotine-treated WT cells exhibited a higher level of ROS compared to the untreated WT cells. Especially a 2.0 g/L-nicotine treatment led to the increase in the level of ROS in nicotine-treated cells, and this level was 24.4 times higher than that in untreated cells. Using H_2_O_2_ as positive control, it was observed that the level of ROS produced by 2.0 g/L-nicotine treatment, is higher than that produced by 2 mM-H_2_O_2_ treatment. Therefore, it can be inferred that the higher the concentration of nicotine, the stronger the oxidative stress induced.

To confirm that QS could contribute to nicotine tolerance by activating antioxidant defense system, the activity of antioxidant enzymes were measured among WT, Δ*lasR*, and Δ*rhlR* strains. As shown in Figure 2B, there was no difference in the activity of CAT among the WT and mutant strains without nicotine stress. The activity of CAT significantly increased on exposure to 1.6 g/L of nicotine in the WT and Δ*rhlR* strains compared to that in the Δ*lasR* strain. Though the CAT activity decreased under a 2.0 g/L-nicotine treatment due to toxicity, the WT strain showed a significantly higher activity of CAT than that observed in Δ*lasR*, and this activity had no significant difference with that observed in Δ*rhlR* strain.

Additionally, we measured the SOD activity among these three strains. However, no significant increase was observed for this parameter (Figure 2C). Taking the above-mentioned data into account, bacterial QS involving the LasIR and RhlIR circuits, regulate the anti-oxidative response to nicotine stress in WT strain. Further studies are required to explain why QS promotes CAT activity, and not SOD activity.

### QS-regulated biofilm formation favored of nicotine tolerance

Biofilm formation, mainly regulated by QS, could be another reason for stress tolerance (Hammer and Bassler, 2003; Daniels et al., 2004; Shrout and Nerenberg, 2012). Compared to planktonic cells, biofilm formation increases stress tolerance up by 10–1,000 folds (Hazan et al., 2016). Another parallel assumption is that QS-regulated biofilm formation is beneficial for nicotine tolerance. Therefore, to clearly understand the effect from QS-regulated biofilm formation on nicotine tolerance, we compared the biofilm formation of WT and Δ*lasR* and Δ*rhlR* strains on exposure to nicotine.

**Figure 3:**
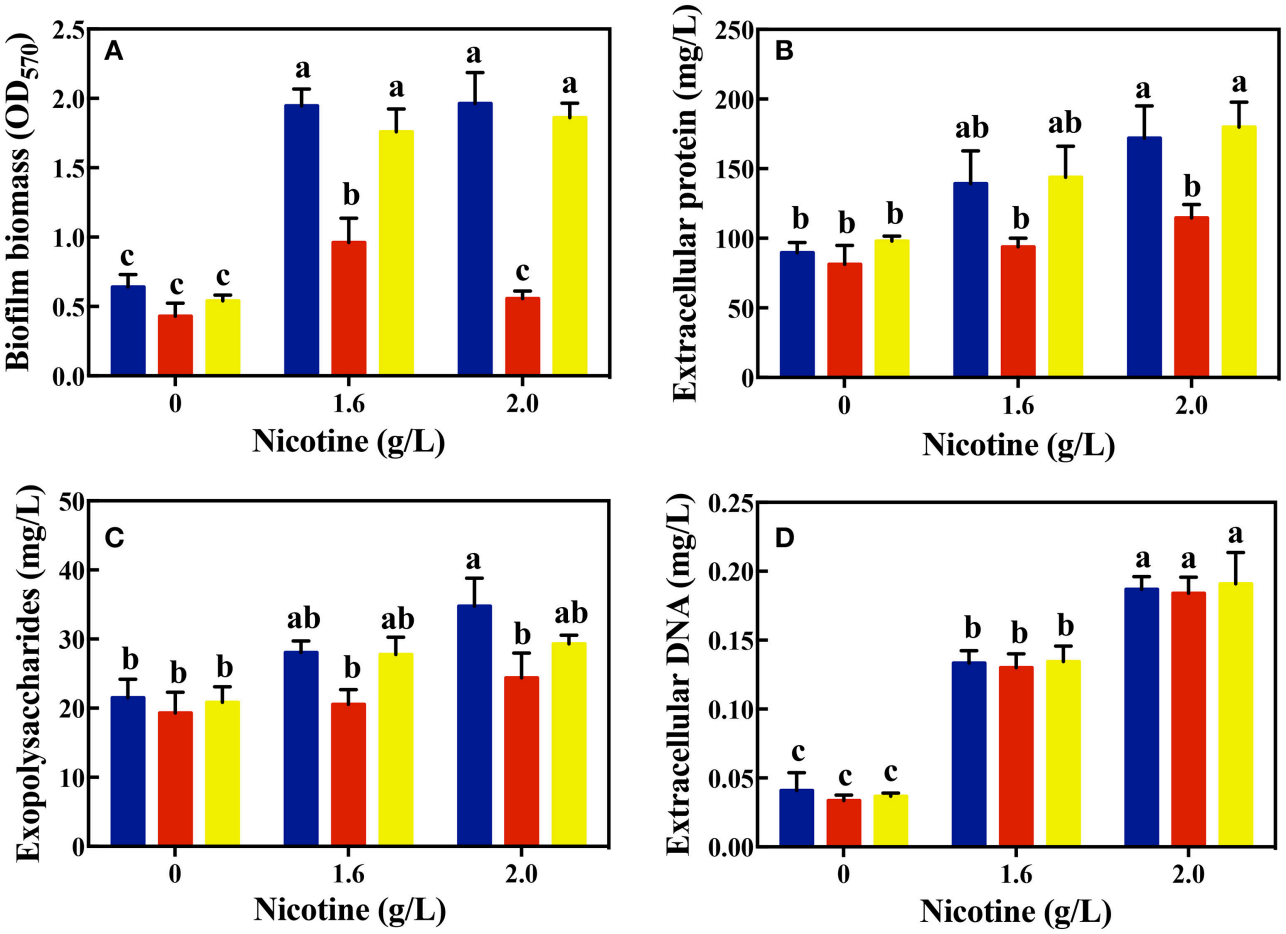
Comparison of biofilm biomass **(A)** and its components: extracellular protein **(B)**, polysaccharides **(C)**, and extracellular DNA **(D)** among different strains (WT, blue bars; Δ*lasR*, red bars; Δ*rhlR*, yellows bars) on exposure to different concentrations of nicotine. Different letters indicate significant difference at *p* < 0.05 and the same letter indicates no significant difference.

As shown in Figure 3A, there was no significant difference in the biofilm formation of WT and Δ*lasR* and Δ*rhlR* strains in absence of nicotine. On treating with 1.6 and 2.0 g/L of nicotine, the biofilm biomass of WT and Δ*rhlR* increased significantly. There was no difference of biofilm biomass between WT and Δ*rhlR*. However, the biofilm biomass of Δ*lasR* was significantly lower than that of the other two strains.

In addition, the amount of certain biofilm components was analyzed. As shown in Figures 3B–D, the level of EPS and extracellular proteins in the biofilms of the WT and Δ*rhlR* strains was significantly higher than that of the Δ*lasR* strains under a 1.6 g/L-nicotine treatment. After exposure to 2.0 g/L of nicotine, no significant difference in the level of EPS between the biofilms of Δ*lasR* and Δ*rhlR* was observed. The level of EPS and extracellular protein in the biofilm of the WT strain was significantly higher than that in the biofilm of Δ*lasR* under a 2.0 g/L-nicotine treatment. The extracellular DNA content was almost equivalent among three strains, indicated by an extremely small amount of extracellular DNA in the biofilm.

**Figure 4:**
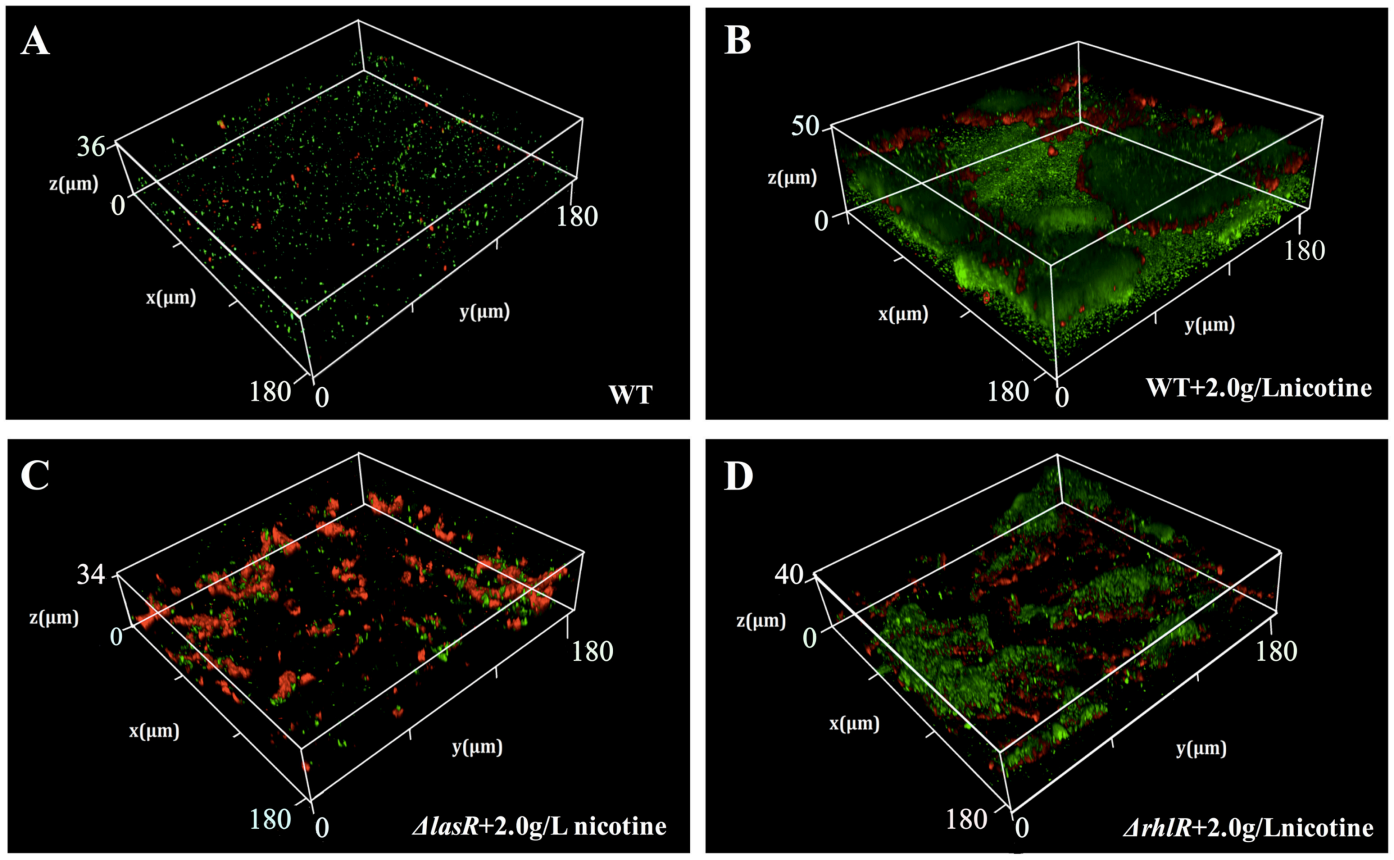
Comparison of biofilm structure and proportion of live/dead cells (green, live cells; red, dead cells) in biofilm among WT **(A)**, WT + 2.0 g/L nicotine **(B)**, Δ*lasR* + 2.0 g/L nicotine **(C)**, and Δ*rhlR* + 2.0 g/L nicotine **(D)**.

Moreover, we used the CLSM to observe the structure of biofilm and employed a double live/dead staining to determine cell viability in biofilm. As shown in Figure 4, the biofilm thickness of WT and Δ*rhlR* strains increased under nicotine stress. However, the biofilm formation of Δ*lasR* was significantly inhibited under nicotine stress. Compared to WT and Δ*rhlR* biofilm, the number of dead cells dramatically increased in the Δ*lasR* biofilm. All above data demonstrated that QS-regulated biofilm formation was also involved in enhancement of nicotine tolerance.

### LasIR being responsible for nicotine tolerance

As seen in Figures 2B, 3A, the CAT activity and biofilm biomass in the Δ*lasR* strain was significant lower than the WT and Δ*rhlR* strain. Meanwhile there were no significant differences for the same parameters between the WT and Δ*rhlR* strains. It suggested that the LasIR circuit played more important role in nicotine tolerance than the RhlIR circuit. Bacteria lacking a functional LasIR circuit, are sensitive to nicotine. To confirm these, competition experiments between the WT and Δ*lasR* strains or between the Δ*rhlR* and Δ*lasR* strains were conducted.

**Figure 5:**
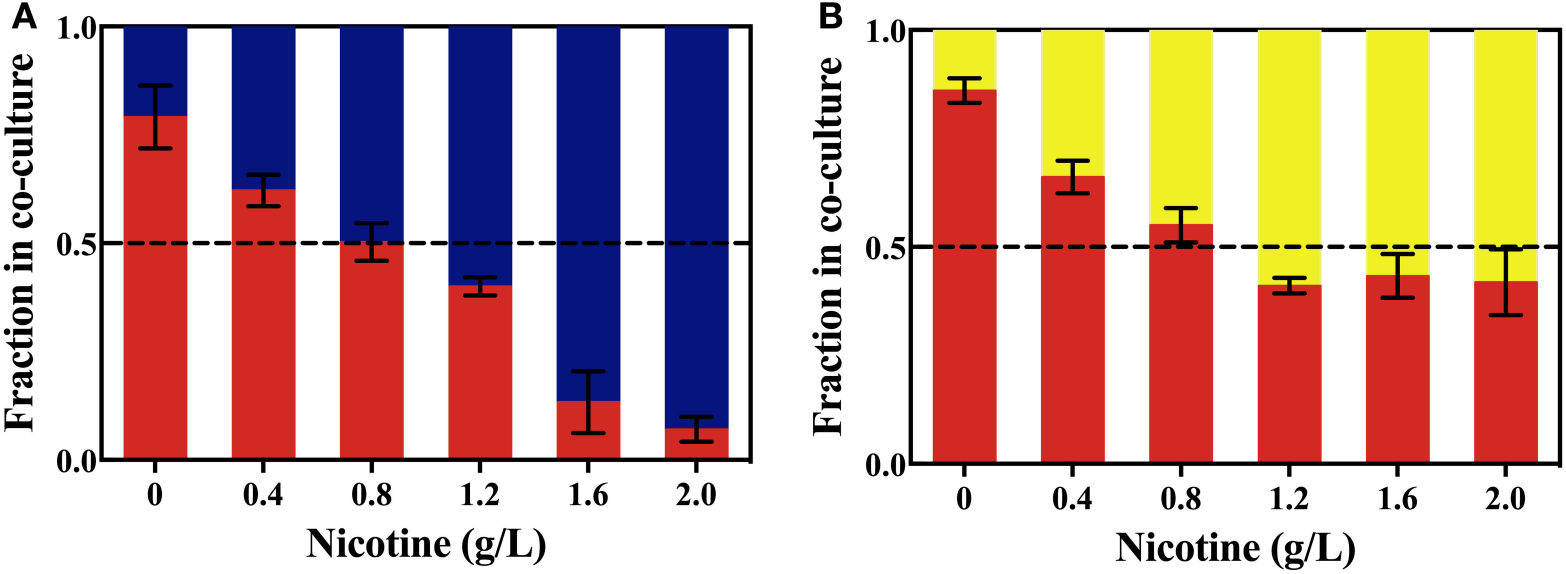
The competition between the WT (blue bars) and Δ*lasR* (red bars) strains **(A)**, or between the Δ*rhlR* (yellows bars) and Δ*lasR* strains **(B)** on exposure to different concentrations of nicotine.

As shown in Figure 5, without nicotine stress, Δ*lasR* growth was higher than that of the WT or Δ*rhlR* strains. After 24 h, 79.1 and 86.1% of the total population in the WT competition system and the Δ*rhlR* competition system, respectively, were Δ*lasR* cells. With the increase in nicotine concentration, the proportion of Δ*lasR* population significantly decreased. It was reduced to 16.7% in WT competition system under 2.0 g/L-nicotine stress. The decrease of Δ*lasR* fitness advantage with the increase of nicotine is consistent with the above hypothesis.

**Figure 6:**
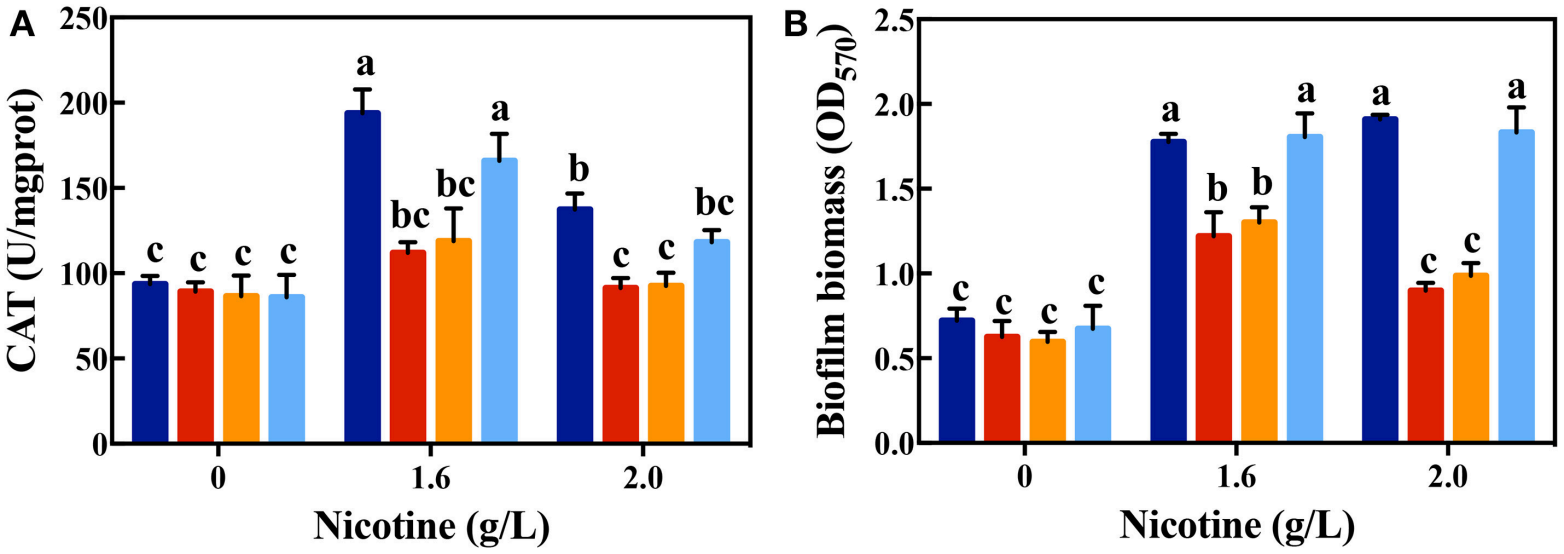
CAT activity **(A)** and biofilm formation **(B)** after complementing Δ*lasI* strain with 3OC12-HSL on exposure to different concentrations of nicotine (WT: dark blue bars; Δ*lasR*: red bars; Δ*lasI*: orange bars; Δ*lasI* + 3OC12-HSL: light blue bars). Different letters indicate significant difference at *p* < 0.05 and the same letter indicates no significant difference.

For the competition experiment, other factors except the nicotine tolerance could affect the advantageous fitness. Thus, Δ*lasI* supplementation with 3OC12-HSL was implemented in further experiments. Δ*lasI* is a signal-deficient mutant, without the ability to synthesize 3OC12-HSL, but with the functional signal receptors, LasR. According to the mechanism of QS, exogenous additional of 3OC12-HSL also could bind to LasR and trigger the expression of the corresponding regulon (Wang et al., 2015). As shown in Figure 6, the CAT activity and biofilm formation in the Δ*lasI* strain was similar to those in the Δ*lasR* strain. However, addition of 3OC12-HSL significantly increased the CAT activity and biofilm formation in the Δ*lasI* strain, and they were nearly identical with those in the WT strain. Both competition systems in coculture and signal complementary assays for Δ*lasI* confirm that LasIR circuit is important for nicotine tolerance in *P. aeruginosa*.

### QQ acting even better under nicotine stress

Quorum quenching (QQ) was widely used for controlling pathogenicity in *P. aeruginosa*, and reducing the level of virulence factors such as elastase, pyocyanin, and pyoverdine (Lee and Zhang, 2015). As the above-mentioned results indicate, QS played important role in nicotine tolerance. A rational deduction was that QQ could act efficiently under nicotine stress. To prove it, the production of elastase, pyocyanin, and pyoverdine was compared with or without QQ treatments.

**Figure 7:**
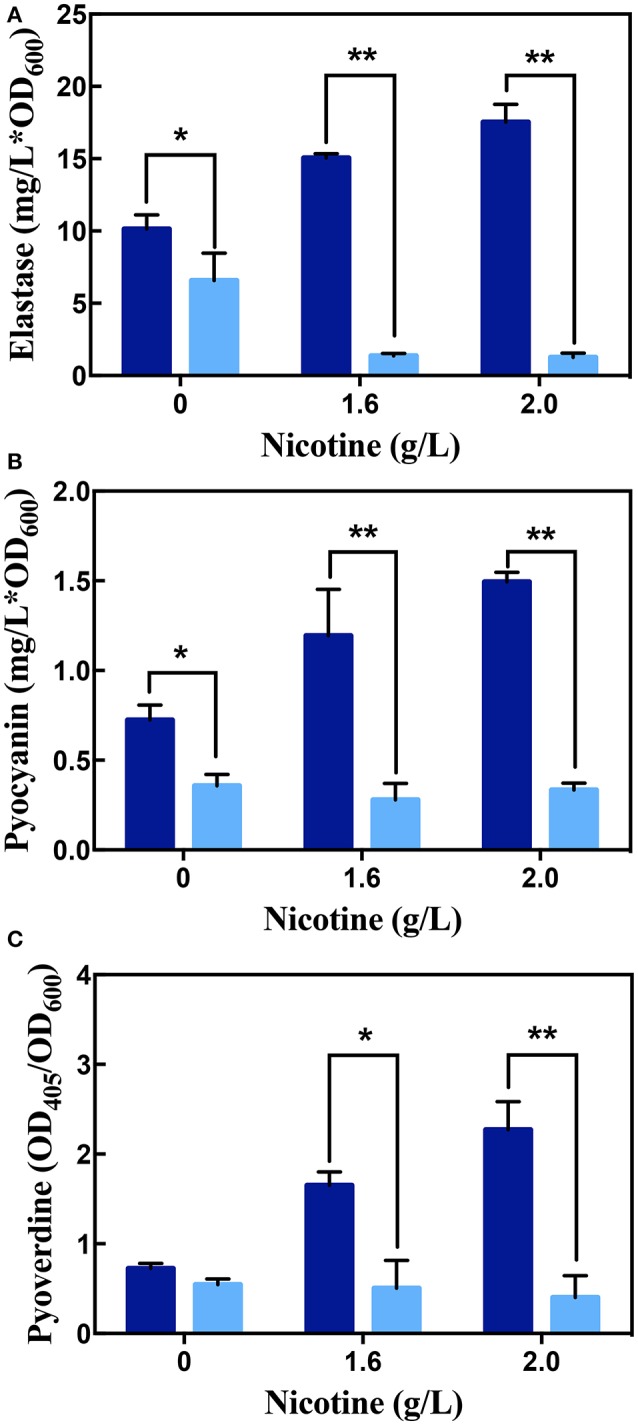
The production of elastase **(A)**, pyocyanin **(B)**, and pyoverdine **(C)** in WT on exposure to different concentrations of nicotine, with (light blue bars) or without (dark blue bars) acylase I. ^**^ and ^*^ indicate significant difference at *p* < 0.01 and *p* < 0.05, respectively.

As seen in Figure 7, along with the increasing of nicotine, the content of elastase, pyocyanin, and pyoverdine enhanced. It suggested that nicotine induces the QS pathway in *P. aeruginosa*. Addition of the acylase I, interrupted these pathways and decreased the production of elastase and pyocyanin. Without nicotine treatments, there was a 35.14 and 43.13% reduction in the level of elastase and pyocyanin after acylase I treatment, respectively, compared to non-addition of the acylase I. There were no significant differences between the level of pyoverdine before and after acylase I treatments.

Under nicotine stress, acylase I significantly decreased the secretion of all virulence factors. After acylase I treatment, the proportion of elastase, pyocyanin, and pyoverdine reduced to 18.23, 23.31, and 30.53% under 1.6 g/L of nicotine, respectively, compared to the levels before the acylase I treatment. After exposure to 2.0 g/L nicotine, the proportion of elastase, pyocyanin, and pyoverdine reduced to 7.13, 22.39, and 17.69%, respectively, compared to the levels before the acylase I treatment. Among all virulence factors, the production of elastase was inhibited the most. Compared to untreated cells, there was a greater decrease for all tested virulence factors under nicotine-treated cells.

## Discussion

The toxicity of nicotine on bacteria, through high permeability in cell membrane, oxidative stress, and macromolecular (protein and DNA) damage, has been well-studied (Huang et al., 2014). In this study, we compared the nicotine tolerance between WT and QS mutant strains, and found that the bacterial growth was significantly inhibited by nicotine if the QS pathway was nonfunctional. In addition, significantly higher CAT activity, biofilm biomass, and number of live cells in biofilm were found for the WT strain than for Δ*lasR*. These results confirmed that QS played an important role in nicotine tolerance. Besides nicotine stress, Walawalkar et al. (2016) showed that QS of *Salmonella typhi* aided in oxidative stress management. According to Lin et al. (2016), DqsIR QS mediated gene regulation of the extremophilic bacterium *Deinococcus radiodurans* in response to oxidative stress. This indicates that QS could protect bacteria from a wide range of stress.

Under nicotine stress, different strains had variant CAT activity. Highest CAT activity was observed in the WT strain, while the lowest in the Δ*lasR* strain. QS controls expression of CAT genes and mediates susceptibility to H_2_O_2_ (Hassett et al., 1999). Compared to individuality, cells in biofilm could help each other to protect themselves from different kinds of stress (Oliveira et al., 2015). Several studies have shown that biofilm development was regulated by QS (Tseng et al., 2016). Moreover, weakening of biofilm structure in *P. aeruginosa* has been linked to the disruption of LasIR circuit (Sunder et al., 2017). From Figure 3A, it can be observed that biofilm biomass increased in nicotine stress when LasIR circuit is functional. Both, antioxidant-production ability and biofilm formation, which are regulated by QS, enhance the nicotine tolerance.

Taking the CAT activity and biofilm biomass into account, LasIR circuit promotes nicotine tolerance rather than the RhlIR circuit. We also conducted competition experiments between the Δ*rhlR* and Δ*lasR* strains. In LB media without nicotine, the Δ*lasR* strain had a significant fitness than the Δ*rhlR* strain. However, with the increase in nicotine concentration in LB media, the growth of the Δ*rhlR* strain increased significantly compared to that of the Δ*lasR* strain (Figure 5). From Figure 6, supplementation of the Δ*lasI* strain with 3OC12-HSL led to the culture showing similar CAT activity and biofilm formation to those of the WT strain, under nicotine stress. Both competition in coculture and signal complementary assays for Δ*lasI* confirmed that LasIR circuit was more important than the RhlIR circuit in the response to nicotine stress.

The members of the QS pathway are promising targets for treatment of pathogenic infection (Köhler et al., 2010). Several QQ reagents have been developed (O'Loughlin et al., 2013). As shown in Figure 7, the inhibition efficiencies of acylase I are different for various of virulence factors. According to the genetic network of the PAO1 strain, *lasR, rhlR*, and *pqsE* have been reported to be involved in the production of pyocyanin (O'Loughlin et al., 2013; Rampioni et al., 2016), while *ampR, ppyR, mexT*, and *lasR* are involved in the production of elastase (Van Delden et al., 1998; Maseda et al., 2004; Kong et al., 2005; Attila et al., 2008). There are much more genes contributing to elastase production than those contributing to pyocyanin production. Thus, the inhibition efficiency for pyocyanin was higher, while less elastase production was inhibited. The production of pyoverdine was regulated by PQS, a type of a QS pathway that is not mediated by AHLs, in *P. aeruginosa* (Lee and Zhang, 2015). Acylase I can only interrupt AHLs-mediated QS (Zhang et al., 2015). Thus, acylase I did not inhibit the production of pyoverdine under no nicotine treatment conditions. Different QS circuits regulate the secretion of different virulence factors (Chugani et al., 2001). One virulence factor is regulated by completely or partially regulated by QS (O'Loughlin et al., 2013; Husain et al., 2017). QQ was successful in reducing the production of certain, but not all, kinds of tested virulence factors in *P. aeruginosa*.

**Figure 8:**
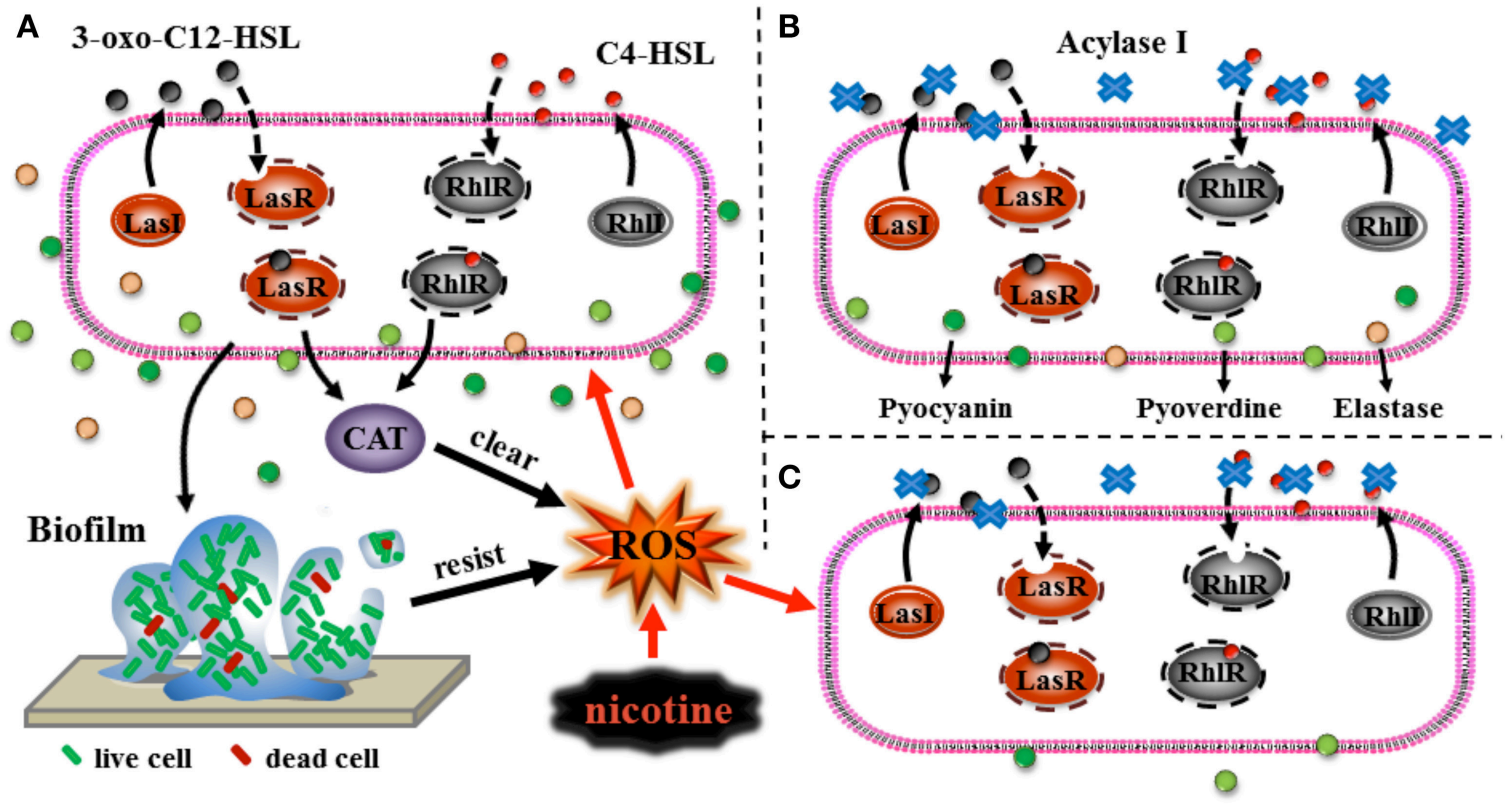
Schematic representations of the role of QS in nicotine tolerance **(A)**, QQ leading to reduction of virulence **(B)**, and the high efficiency of QQ under nicotine exposure leading to reduction of virulence **(C)**. Black arrows represent the direction of signals transduction or direction of transport of the virulence factors, black dotted arrows represent the process of the binding of ligands to the respective receptors, black thick arrows represent clearing of ROS or resistance to stress, and red arrows represent induction of ROS production or induction of stress to cells.

Various conditions, such as pH and temperature, possibly affect the application of QQ in pathogenicity control. pH and temperature could affect the existence of QS signal in the environment (Yates et al., 2002). Few studies have focused on the efficiency of QQ under stress. In this study, the QQ showed a higher efficiency in decreasing the production of virulence factors, including elastase, pyocyanin, and pyoverdine under nicotine stress compared to no stress. Nicotine is toxic to most kinds of bacteria. QS contributes to nicotine tolerance (Figure 8A). Interruption of QS led to the decrease in both, nicotine tolerance and virulence (Figures 8B,C). After loss of nicotine tolerance, the bacterial population possibly reduces their virulence in order to survive as a trade-off. Though we can not apply of QQ under nicotine stress due to its addiction, it gives us an explanation that the combination of QQ with antibiotics is higher efficient than only one treatment (Wang et al., 2018). Therefore, this study not only improves our understanding regarding the role of QS in environmental stress tolerance, but also provides a foundation for the development of QQ-based strategies to control or reduce the pathogenicity of bacteria (Figure 8).

## Author contributions

MW, HT, and DS conceived and designed the experiments. HT, YZ, YM, and MT performed the experiments. HT and MW analyzed the data. MW and DS contributed reagents, materials, and analysis tools. MW and HT wrote the paper.

### Conflict of interest statement

The authors declare that the research was conducted in the absence of any commercial or financial relationships that could be construed as a potential conflict of interest. The reviewer MC-Y and handling Editor declared their shared affiliation.

## Abbreviations

CAT: Catalase
CV: Crystal violet
EPS: Extracellular polymeric substances
LB: Luria-Bertani
3OC12-HSL: *N*-3-oxo-dodecanoyl homoserine lactone
OD: Optical density
QQ: Quorum quenching
QS: Quorum sensing
ROS: Reactive oxygen species
SOD: Superoxide dismutase
TNBSA: Trinitrobenzene sulfonic acid
WT: Wildtype.
